# X-Linked Agammaglobulinemia Presenting with Secondary Hemophagocytic Syndrome: A Case Report

**DOI:** 10.1155/2013/742795

**Published:** 2013-01-21

**Authors:** Can Ozturk, Sumer Sutcuoglu, Berna Atabay, Afig Berdeli

**Affiliations:** ^1^Department of Pediatrics, Izmir Tepecik Education and Research Hospital, Ministry of Health, 35100 Izmir, Turkey; ^2^Department of Pediatrics, Molecular Medicine Laboratory, Medical School, Ege University, 35040 Izmir, Turkey

## Abstract

*Introduction*. Coincidence of X-linked agammaglobulinemia (XLA) and secondary hemophagocytic syndrome (sHS) is atypical. Both diseases are rare and pathogenesis of the latter one is not clearly known. *Case Presentation*. A 5-year-old boy was diagnosed both with XLA and sHS. However, in his history, he did not have severe and recurrent infections. Bruton tyrosine kinase (BTK) gene mutation was present (c.1581_1584delTTTG). To the best of the authors' knowledge, coincidence of XLA and sHS had not been reported in the literature before. *Conclusion*. Patients with XLA are extremely vulnerable to recurrent bacterial infections. The diagnosis of XLA with sHS at any time of life is both an interesting and challenging situation without history of recurrent bacterial infections.

## 1. Introduction

The defect in Bruton's tyrosine kinase (BTK) is caused by the absence of mature B cells in peripheral blood. Normally, patients with XLA is exposed to bacterial infections in the early childhood and live vaccines can be harmful. Furthermore, what distinguishes XLA from most other humoral defects is the increased frequency of chronic enteroviral infections [1].

Hemophagocytic syndrome (HS) is a life-threatening disorder and is uncommon in children. HS can be divided into two groups: the familial (genetic) form and the secondary (reactive) form. The perforin 1 gene (PRF1), hMunc13-4 gene (UNC13D), STX11, and RAB27A are defined as genetic defects in the familial form. The secondary form can be due to three main etiologies: infections, malignancies, and systemic connective tissue disorders. In older children, HS is often associated with the secondary form caused by viral infections [2].

We here present a 5-year-old case of XLA who suffered from severe pneumonia. He was diagnosed XLA shortly after sHS diagnosis. A mutation (c.1581_1584delTTTG) in BTK gene was present. This mutation is reported for the second time in the literature according to our knowledge.

## 2. Case Presentation

The 5-year-old male child had severe pneumonia and pleural effusion when he was admitted to our hospital. His body temperature was constantly high in the last two months despite the peroral antibiotic treatment. His parents were not consanguineous. 

History was not remarkable with respect to recurrent infections or frequent hospitalizations. We found out that in pedigree chart, his brother, one of his maternal uncles, and his maternal aunt's two sons died because of infections in early infancy. He had an obvious right foot drop and muscle wasting in his right leg which developed after he was administered oral polio vaccine when he was 1.5 years old. 

On his physical examination, his weight was in the 50th percentile and his height was in the 75th percentile, and axillary temperature was 39-40°C. There were no visible tonsils and palpable lymph nodes. Breath sounds were notably diminished in the left lung. He had no hepatomegaly and splenomegaly but he met the diagnostic criteria of sHS (Table 1). 

**Figure 1:**
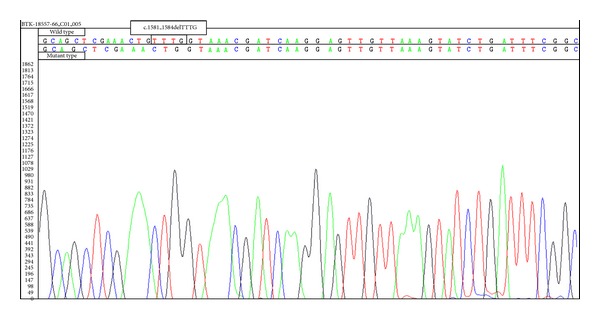
BTK mutation of the patient (c.1581_1584delTTTG).

Initial laboratory investigations are summarized in Table 1. Anemia, neutropenia, thrombocytopenia, hypertriglyceridemia, and hyperferritinemia were found in laboratory studies. Bone marrow aspiration revealed hemophagocytosis. Serum immunoglobulin levels were extremely low (IgG: <33 mg/dL, IgM: 12.6 mg/dL, and IgA: <6.67 mg/dL). Reference values of serum immunoglobulin for 5-year-old Turkish children are as follows: IgG: 528–1490 mg/dL, IgM: 33–207 mg/dL, and IgA: 23–205 mg/dL [3]. Flow cytometry analyses demonstrated the complete lack of circulating B cells (CD19% 0.5) (Table 1). In his healthy Turkish peers, the range of the normal lymphocyte values are given in Table 1 (% CD19 11–31) [4]. Mutations of perforin and SH2D1A genes were not detected, whereas BTK gene mutation was present (c.1581_1584delTTTG) (Figure 1). 

The treatment was begun on January 21, 2010 and the patient was administered intravenous teicoplanin 20 mg/kg/day and ceftriaxone 100 mg/kg/day for 21 days; however, his temperature did not drop. No infectious agents were isolated from the cultures of blood, stool, and other body fluids drawn on the onset of the treatment and throughout the treatment. His bone marrow analysis performed on the 21st day of the treatment showed HS. Therefore, intravenous immune globulin (1.0 g/kg/day) for two days was administered. Anemia, neutropenia, thrombocytopenia, and hypofibrinogenemia were improved following the administration of intravenous immune globulin and he was discharged on February 22, 2010. 

## 3. Discussion

X-linked agammaglobulinemia is a hereditary immunodeficiency caused by mutations in the gene encoding BTK and is characterized by recurrent severe infections. These recurrent infections occur at about 7–9 months after birth, when transplacental antibody titers begin to decrease and when the infant's body is unable to compensate for low levels of antibodies. The diagnosis of XLA is based on lymphoid hypoplasia, which is realized during the physical examination, low serum immunoglobulin, the absence of specific antibody response, and the lack of B cells in peripheral blood. Some patients may develop vaccine-related paralytic poliomyelitis [5, 6]. The patient had all the findings of XLA except frequent infections.

Our patient was diagnosed with XLA shortly after sHS diagnosis. Splenomegaly and hepatomegaly, which are progressive, are usually present in sHS, but physical examination revealed none of them in our patient [7]. In sHS, some patients may have lymph nodes enlargement, although the lymph nodes of our patient were not palpable. However, adenoids and tonsils in XLA disease are frequently rudimentary and lymph nodes are reduced in size. Coincidence of XLA and sHS has not been reported in the literature [7]. Our patient is the first case who had an unusual association between XLA and sHS.

Since secondary HS is rare in older children and mostly develops due to infections and symptoms of familial HS generally begin during infancy and early childhood, familial HS was the prediagnosis. The review of the literature revealed only one familial HS report [8]. Immunosuppressive therapy, as well as anticytokinic therapy including TNF-blockade may be employed for cytokine storm in HS [9]. As our patient's brother and cousins died, SH2D1A and perforin genes were investigated only in our patient, but no mutations were detected. 

The patient was diagnosed as XLA, since mutation was found in BTK gene. Analysis of the patient's DNA revealed the deletion of 4 nucleotides (TTTG) in exon 16 of the BTK gene (c.1581_1584delTTTG). This mutation is reported for the second time in the literature. The mutation (c.1581_1584delTTTG) of BTK gene was previously reported in the literature by Ameratunga et al. [10] for the first time. Similarly, they did not report history of recurrent infections, either. Genotype and phenotype correlations have not been established in patients with XLA currently. A very few publications are available reporting this paradoxical clinical and laboratory findings [11, 12]. 

This mutation in XLA may result in delayed recurrence of infections and is of importance. We wanted to point out to the coexistence of XLA and sHS which leads to unusual lymphoid hypoplasia.

## 4. Conclusion

Clinicians should be aware that primary immune deficiencies at any time of the childhood may manifest with an atypical course. XLA is a disorder that is extremely susceptible to bacterial infections and presents with recurrent infections. The recognition of XLA with sHS at any time of life is a rare and challenging condition without history of recurrent infections.

## Figures and Tables

**Table 1:** Laboratory characteristics of the patient.

	Presentation January 21, 2010	Diagnosis of HS February 09, 2010	After treatment February 22, 2010	Reference
WBC (ANC)	10.6	0.8	6.0	2.0–7.5 × 10^9^/L
Hemoglobin	9.7	8.7	9.3	14–16 g/dL
Platelets	592	20	634	150–450 × 10^9^/L
INR	0.86	1.4	0.83	0.8–1.2
PTT	13.4	30.9	32.7	22.6–35 sec
Fibrinogen		241		175–400 mg/dL
AST	18	96	27	0–35 U/L
ALT	10	69	20	0–45 U/L
Ferritin	80	562	65	7–142 ng/mL
LDH	335	798	272	110–295 U/L
Triglycerides	79	500	153	30–199 mg/dL
IgG	<33.3		1400	528–1490 mg/dL*
IgM	12.6		10.5	33–207 mg/dL*
IgA	<6.67		<6.67	23–205 mg/dL*
CD3%	94		72	55–79*
CD19%	0.5		0.46	11–31*
CD16+56%	7		8	5–28*

*normal values for Turkish children are presented in [3, 4].
